# Youth Healthy Eating Index (YHEI) and Diet Adequacy in Relation to Country-Specific National Dietary Recommendations in Children and Adolescents in Five Mediterranean Countries from the DELICIOUS Project

**DOI:** 10.3390/nu16223907

**Published:** 2024-11-15

**Authors:** Francesca Giampieri, Alice Rosi, Francesca Scazzina, Evelyn Frias-Toral, Osama Abdelkarim, Mohamed Aly, Raynier Zambrano-Villacres, Juancho Pons, Laura Vázquez-Araújo, Sandra Sumalla Cano, Iñaki Elio, Lorenzo Monasta, Ana Mata, María Isabel Pardo, Pablo Busó, Giuseppe Grosso

**Affiliations:** 1 Department of Clinical Sciences, Università Politecnica delle Marche, 60131 Ancona, Italy; 2 Research Group on Food, Nutritional Biochemistry and Health, Universidad Europea del Atlántico, Isabel Torres 21, 39011 Santander, Spain; 3 Joint Laboratory on Food Science, Nutrition, and Intelligent Processing of Foods, Polytechnic University of Marche, Italy, Universidad Europea del Atlántico Spain and Jiangsu University, China, 60131 Ancona, Italy; 4 International Research Center for Food Nutrition and Safety, Jiangsu University, Zhenijang 212013, China; 5 Human Nutrition Unit, Department of Food and Drug, University of Parma, 43124 Parma, Italy; 6 School of Medicine, Universidad Católica de Santiago de Guayaquil, Av. Pdte. Carlos Julio Arosemena Tola, Guayaquil 090615, Ecuador; 7 Faculty of Physical Education, Assiut University, Assiut 71515, Egypt; 8 Escuela de Nutricíon y Dietética, Universidad Espíritu Santo, Samborondón 0901952, Ecuador; 9 Editorial Luis Vives (EDELVIVES), Carretera de Madrid, 50012 Zaragoza, Spain; 10 BCC Innovation, Technology Center in Gastronomy, Basque Culinary Center, 20009 Donostia-San Sebastián, Spain; 11 Basque Culinary Center, Faculty of Gastronomic Sciences, Mondragon Unibertsitatea, 20009 Donostia-San Sebastián, Spain; 12 Department of Health, Nutrition and Sport, Universidad Internacional Iberoamericana, Campeche 24560, Mexico; 13 Faculty of Health Sciences, Universidad de La Romana, La Romana 22000, Dominican Republic; 14 Fundación Universitaria Internacional de Colombia, Bogotá 111321, Colombia; 15 Institute for Maternal and Child Health, IRCCS Burlo Garofolo, 34137 Trieste, Italy; 16 Technological Institute for Children’s Products & Leisure AIJU, 03440 Alicante, Spain; 17 Department of Biomedical and Biotechnological Sciences, University of Catania, 95123 Catania, Italy; 18 Center for Human Nutrition and Mediterranean Foods (NUTREA), University of Catania, 95123 Catania, Italy

**Keywords:** dietary recommendations, diet quality, Mediterranean diet, children, adolescents

## Abstract

Background/Objectives: The diet quality of younger individuals is decreasing globally, with alarming trends also in the Mediterranean region. The aim of this study was to assess diet quality and adequacy in relation to country-specific dietary recommendations for children and adolescents living in the Mediterranean area. Methods: A cross-sectional survey was conducted of 2011 parents of the target population participating in the DELICIOUS EU-PRIMA project. Dietary data and cross-references with food-based recommendations and the application of the youth healthy eating index (YHEI) was assessed through 24 h recalls and food frequency questionnaires. Results: Adherence to recommendations on plant-based foods was low (less than ∼20%), including fruit and vegetables adequacy in all countries, legume adequacy in all countries except for Italy, and cereal adequacy in all countries except for Portugal. For animal products and dietary fats, the adequacy in relation to the national food-based dietary recommendations was slightly better (∼40% on average) in most countries, although the Eastern countries reported worse rates. Higher scores on the YHEI predicted adequacy in relation to vegetables (except Egypt), fruit (except Lebanon), cereals (except Spain), and legumes (except Spain) in most countries. Younger children (*p* < 0.005) reporting having 8–10 h adequate sleep duration (*p* < 0.001), <2 h/day screen time (*p* < 0.001), and a medium/high physical activity level (*p* < 0.001) displayed a better diet quality. Moreover, older respondents (*p* < 0.001) with a medium/high educational level (*p* = 0.001) and living with a partner (*p* = 0.003) reported that their children had a better diet quality. Conclusions: Plant-based food groups, including fruit, vegetables, legumes, and even (whole-grain) cereals are underrepresented in the diets of Mediterranean children and adolescents. Moreover, the adequate consumption of other important dietary components, such as milk and dairy products, is rather disregarded, leading to substantially suboptimal diets and poor adequacy in relation to dietary guidelines.

## 1. Introduction

Diet has been established as a major determinant of long-term health globally, contributing to a substantial share of years lived with disability due to disease worldwide [1]. Nutritional factors have been estimated to be responsible for a large share of disability and mortality due to non-communicable diseases [2]. However, when exploring children’s and adolescents’ diets, the current evidence is not encouraging [3]: there is a growing trend towards a worsening of diet quality in favor of so-called “Westernized” dietary habits, characterized by a lower intake of minimally processed plant-based foods in favor of nutrient-poor, highly energy-dense foods with an unfavorable nutrient content (low fiber and protein and high sugar, sodium, and saturated and trans fatty acids, among others) [4,5]. Such dietary features represent a known burden in certain countries, such as the US, the UK, and Australia [6,7,8,9]; however, over the last few decades, a higher adoption of unhealthy diets have also been recorded in Mediterranean countries, where an abandonment of traditional dietary patterns (such as the Mediterranean diet) has been accompanied by rising trends in obesity rates, especially among younger generations [10,11,12]. The traditional Mediterranean dietary pattern does not comprise identical foods across all countries facing the Mediterranean sea, but it has some common features, including the high consumption of fruits, vegetables, legumes, nuts, and whole grains; the moderate intake of fish and poultry; the low consumption of red meat and sweets; the use of olive oil as the primary fat source; and a variety of underrated, yet important, features that should be mentioned, including seasonality, the use of spices and herbs, and the moderate consumption of various dairy products and eggs, as well as fermented foods and wine (among adults) [13]. This dietary pattern not only offers nutritional benefits but also encompasses cultural and lifestyle aspects, emphasizing social interactions and physical activity, as well as preserving the environment and promoting a circular economy [14]. Current evidence suggests that Mediterranean countries from Southern Europe [15,16,17] and North Africa/the Middle East [18,19,20] are facing a cultural change due to the introduction of more “globalized” diets leading to a slow abandonment of culturally acceptable, traditional dietary patterns [21]. In such a geographic area, rates of adherence to a traditional diet have been reported to have significantly dropped over time [22]. Notably, the abandonment of the Mediterranean diet has been shown to be accompanied by a decrease in diet quality [23,24] while growing trends of childhood obesity are substantially rising, especially in those regions with such an observed worsening [25].

Dietary habits established during childhood and adolescence have profound effects on individual’s immediate health, growth, and development, as well as long-term outcomes related to chronic disease risk and overall well-being [26]. Adopting adequate diets has been widely promoted for its relation to health, including reduced risks of obesity and metabolic disorders [27], as well as an association with better sleep quality [28] and academic performance [29], among others. The optimal content of nutrients and potentially protective phytochemicals is considered a key for chronic non-communicable disease prevention [30,31]. In this context, adequacy in relation to dietary recommendations should provide the optimal nutrient intake required for the prevention of deficiency and a reduced risk of the chronic non-communicable diseases associated with dietary risk [32]. While nutritional adequacy aims to explore the sufficient intake of specific essential nutrients, neither the real intake nor the specific requirements for one individual (or population) is truly known; hence, dietary recommendations based on major food groups represent a crucial option to provide an easily accessible message for the general population and achieve better health for all. However, despite its recognized benefits, diet quality among children and adolescents in the Mediterranean basin is rapidly evolving and recently reported to be suboptimal [33]. With the objective to investigate the shortcomings of the implementation and guidelines of nutritional intake, this study aims to explore adequacy in relation to national-specific dietary recommendations and the level of adherence to the Mediterranean diet in Spain, Egypt, Italy, Lebanon, and Portugal, countries participating in the DELICIOUS project.

## 2. Materials and Methods

### 2.1. Study Design and Population

The present study is based on a cross-sectional analysis carried out as part of the European-funded DELICIOUS (unDErstanding consumer food choices and promotion of healthy and sustainable Mediterranean Diet and LIfestyle in Children and adolescents through behavIOUral change actionS) project [34]. Briefly, the project aims to improve adherence to the Mediterranean diet and healthy active lifestyles through the adoption of various initiatives, some already accomplished [35]. Preliminary to this multifaceted intervention study and in line with its main general aims, a survey was conducted to explore the dietary habits of children and adolescents aged between 6 and 17 years from five Mediterranean countries (Italy, Spain, Portugal, Egypt, and Lebanon). The respondents were parents voluntarily included within the network of collaborators of the Technological Institute for Children’s Products and Leisure (AIJU). Based on the recent literature aiming at the same purposes in Mediterranean countries [36,37,38,39,40], a convenience sample of 400 individuals per each Mediterranean country was set. The data collection was carried out via an electronic survey, and a total of 2011 agreed to be enrolled in the survey (a participation rate of 79%), accepting to respond to the tools administered. All the procedures were carried out in accordance with the Declaration of Helsinki (1989) of the World Medical Association, and all participants signed an informed consent form before participating in the study.

### 2.2. Background Variables

Data regarding demographic and lifestyle characteristics were collected. The children’s physical activity level was assessed using the International Physical Activity Questionnaire Short Form (IPAQs), which reports the level of physical activity, referring to the last 7 days before compilation, concerning three specific types of activities (walking, moderate-intensity activities, and vigorous-intensity activities); frequency (measured in days per week) and duration (time per day) are collected separately for each specific type of activity [41]. Participants were also asked how many hours, on average, their children are sleeping per day [categorized as (i) <8 h, (ii) 8–10 h, and (iii) >10 h according to the National Sleep Foundation recommendations for school-age children and teenagers] [42], and how many hours they spend on screen, categorized as (i) <2 h/day, (ii) 2–4 h/day, and (iii) >4 h/day. Parents were asked about their educational level [categorized as (i) low (primary), (ii) medium (secondary), and (iii) high (tertiary)] and questions about their family income, family status (i.e., living alone, with a partner, or other family members), and area of living (rural or urban).

### 2.3. Dietary Intake

Daily food intake was recorded by asking parents what their children had eaten in the previous 24 h (with multiple response options by eating occasion and an open blank option to eventually include additional foods). The average intake of food per week was recorded through food frequency questions on the main food groups of interest. Information on the previous day’s intake and answers from the frequency consumption on a daily/weekly basis were harmonized and used to evaluate the adequacy of major food group consumption (e.g., vegetables, fruit, cereals, dairy, meat, legumes, fish, nuts, whole grains, sweets) in relation to national dietary recommendations. The categories used to express the frequency of consumption for all nations were never, 1–2 portions, or ≥3 portions per day or week according to food groups.

### 2.4. Country-Specific Dietary Recommendations

Food-based dietary recommendations were retrieved for each country based on available resources for Italy [43,44], Portugal [45,46], Spain [47,48], Lebanon [49], and Egypt [50]. Most guidelines provided recommendations on fruit and vegetables (altogether for Spain), specifically on meat (Spain and Italy), meat and eggs (Egypt), and protein sources (Lebanon and Portugal) as well as on olive oil (Italy), fats and oils (Portugal), or fats and sweets (Egypt and Lebanon). Other food groups subject to specific recommendations were cereals, legumes, fish and seafood, and dairy products. Since specific guidelines for children and adolescents were not available, dietary recommendations for adults were considered, assuming portion sizes proportional to the reference age.

### 2.5. Diet Quality Evaluation

To evaluate a quantitative measure of diet quality, the Youth Healthy Eating Index (YHEI) [51] was applied to the dietary data obtained. This scoring system addresses the diet quality of older children and adolescents, aiming to establish the consumption of dietary fats, fiber, sodium, and added sugars by examining food choices rather than by direct calculation, resulting in a versatile and easily applicable tool to be applied with younger individuals. The index consists of 13 items, including the consumption of whole-grains, fruit and vegetables, dairy products, meat, snacks, sodas and drinks, margarine and butter, fried foods, and visible animal fats, the use of multivitamins, breakfast eating, and dinners with family, which contribute to a score ranging from 0 to 100. However, for the purposes of the present study, data on the intake of multivitamins and visible fats were not assessed, leading to a maximum possible score of 90, with higher scores reflecting a better diet quality.

### 2.6. Statistical Analysis

Categorical variables are presented as frequencies and percentages, with differences between groups being tested through the Chi-square test. Continuous variables are presented as means and standard deviations (SDs), with differences between groups being tested through an ANOVA test. Logistic regression analyses were performed to calculate the odds ratios (ORs) and 95% confidence intervals (CIs) of the associations between a 1-SD increase in YHEI scores and individual country-specific food-based dietary recommendations.

## 3. Results

The daily/weekly consumption of the main food groups by country is presented in Table 1. For all food groups investigated, there were significant differences in frequency of consumption across countries. Italy, Spain, and Lebanon reported among the highest percentages of a more frequent consumption of fruit and vegetables (12.7% to 14.8% reporting consuming ≥3 portion/d), Lebanon the most frequent consumption of cereals and dairy products (25.6% and 40.5%, respectively, compared to about 10–20% for other countries), as well as nuts (14.8% versus less than about 10% for other countries), Portugal and Italy the most frequent consumption of meat (over 50% versus about 30% for other countries), Egypt the most frequent consumption of legumes (44% versus less than 30% for other countries), Portugal the most frequent consumption of fish and whole grains (over 40% versus less than 30% for other countries), and Spain, Egypt, and Lebanon the most frequent consumption of sweets.

The average scores of the YHEI by country were as follows: 50.2 ± 10.5 in Spain, 49.2 ± 11.5 in Egypt, 55.1 ± 11.5 in Italy, 48.8 ± 10.6 in Lebanon, and 56.1 ± 11.9 in Portugal (*p* < 0.001). The mean scores for specific children’s and parents’ background characteristics are shown in Table 2 and Table 3, respectively. There were significant differences according to several background variables: specifically, children with an adequate sleep duration, shorter screen time, and medium/higher physical activity reported having a higher diet quality. These differences were more evident in some countries; for instance, a higher diet quality in Italian and Portuguese children, higher scores consistently reported in all countries among those with adequate sleep duration (and longer in Portugal), <2 h screen time (although only significant in Italy), and a medium physical activity level (although only significant in Italy and Portugal) (Table 2).

Concerning children’s and adolescents’ YHEI scores by parents’ characteristics, the scores for parent age, higher education, and living with a partner were significantly higher than their counterparts. When exploring diet quality across countries, these trends in education were significant in Egypt, Italy, and Portugal (Table 3).

**Figure 1:**
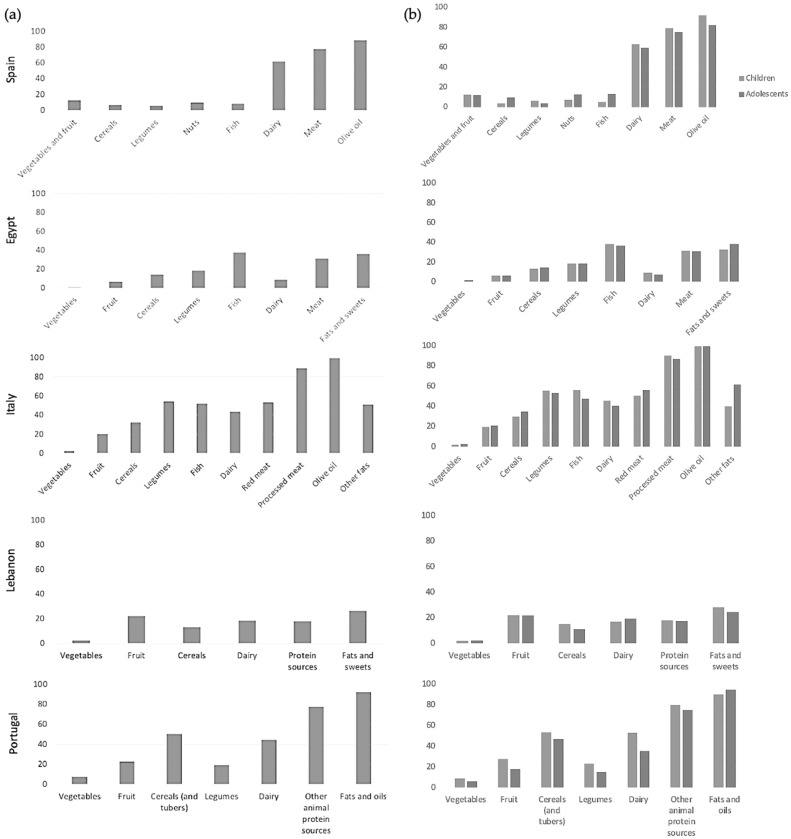
Percentage of adherence to country-specific national dietary recommendations for Spain, Egypt, Italy, Lebanon, and Portugal, total national sample (**a**) and by age groups (**b**).

The main results concerning the level of adherence to country-specific national dietary recommendations for each country are presented in Figure 1. In general, individuals recruited in Eastern Mediterranean countries reported an overall lower adherence to country-specific national dietary recommendations. Overall, adherence to recommendations for certain food groups, such as daily vegetables, fruit, cereals, and legumes (with minor exceptions) were generally low and far from optimal (only a few percent of the sample actually met the recommendations). About half of respondents’ children from Spain, Portugal, and Italy adhered to dairy- and meat-related recommendations, showing higher rates than Egypt and Lebanon. Also, adherence to recommendations on oils (i.e., olive oil) and fats was much higher in Western Mediterranean countries (up to 90% of respondents and over). The analyses stratified by age groups revealed only few differences between children and adolescents in certain countries, although clear trends could not be identified (Figure 1). For instance, Spanish children and adolescents were adhering less than their counterparts to recommendations on cereals, nuts, and fish, and animal foods respectively; however, this trend was not observed in other countries. Italian children adhered more than adolescents to recommendations on fish, dairy, and processed meats but not red meat and fats, while Portuguese ones were generally more in line than adolescents with all recommendations (except for fats and oils).

**Figure 2:**
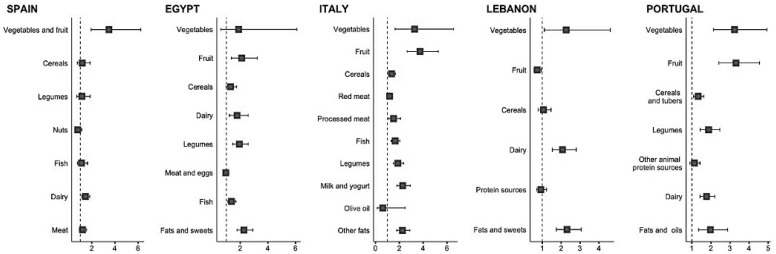
Association between 1-SD increase in diet quality score and adequacy in relation to individual country-specific dietary recommendations.

Figure 2 shows the graphical representation of the probability (ORs and 95% CI) of adequacy in relation to individual country-specific dietary recommendations for each 1-SD increase of the YHEI. The diet quality score did not unequivocally predict all dietary recommendations, and differences also occurred by country. For instance, a 1-SD increase in the score was associated with adequacy in relation to vegetable intake in all countries except in Egypt, while it predicted adequacy to fruit intake in Egypt, Italy, and Portugal but not in Spain and Lebanon. There was a certain consistency of association between increasing diet quality scores and recommendations on dairy products and fats. No associations for meat and other animal protein sources were found in all countries.

## 4. Discussion

In this study, adequacy in relation to country-specific dietary recommendations as well as parameters of the diet quality of children and adolescents were investigated. In general, adherence to recommendations on plant-based foods was low (less than ~20%), including fruit and vegetables adequacy in all countries, legume adequacy in all countries except for Italy, and cereal adequacy in all countries except for Portugal. For animal products and dietary fats, the adequacy to the national food-based dietary recommendations was slightly better (~40% on average) in most countries, although the Eastern countries reported worse rates. We can hypothesize that the lower adherence reported for Egypt and Lebanon may depend on the fact that in their country-specific national guidelines there is no univocal component related to olive oil but a more general indication on fat and sweets consumption (the second component of which is potentially responsible for their poor adherence, hence depending on a higher intake of sweets rather than a low consumption of olive oil). These results also reflected slightly lower diet quality scores for such countries compared to others, albeit the overall diet quality was deemed as largely disappointing.

The findings reported in this study are substantially in line with trends observed over the last decades. An analysis of the Global Dietary Database reported that the mean animal-source food consumption (as an indicator of nutritional adequacy in developing countries) was 1.9 servings per day, representing 16% of children consuming at least three daily servings, with the highest rates in Russia, Brazil, Mexico, and Turkey, and the lowest in Uganda, India, Kenya, and Bangladesh [52]. The results from the aforementioned study reflect adequacy in relation to dietary recommendations concerning dairy products more frequently in children than adolescents (especially in high-income countries) and a general relatively lower intake of fish and seafoods, actually more consumed in Central/Eastern Europe, Central Asia, the Middle East, and North Africa than South/East Asia, Sub-Saharan Africa, and Latin America [52]. Based on recent reports at a global level, the frequency of daily fruit and vegetable consumption is generally low, with an average of about 1.5 portions of fruit and vegetables, and 34.5% and 20.6% reporting having less than a portion fruit and vegetable per day, respectively [53]. These observed trends are mostly unchanged in the Western Pacific and Eastern Mediterranean WHO regions but generally worse when considering children and adolescents from the Americas [53]. When comparing a previous report from 10 European countries (including Mediterranean countries such as Greece, Italy, and Spain, among others) participating in the HELENA (Healthy Lifestyle in Europe by Nutrition in Adolescence) study, the whole international sample showed a higher proportion of agreement with recommendations for fruit and vegetables (around 30–50% depending on sex and age groups) than that reported in our study, while the excessive consumption of meat products and poorer intake of milk and dairy products were also reported, mirroring the trends found in our study [54]. Another investigation conducted on children from 236 schools across Europe reported that, depending on country and sex, only 23% of participants met the WHO recommendation for fruit and vegetables [55]. Other older pan-European reports showed substantially similar trends, with a low consumption of plant-based foods, such as fruit and vegetables, accompanied by the excessive consumption of meat products [56]. Also, trends over time are not favorable, confirming the lower rates we reported in the present report in adolescents [57] and highlighting critically high rates of 6–9 year old children not eating fresh fruit (up to 70% in Spain, and around 30% in Italy and Portugal) and vegetables (90% in Spain, and around 50% in Italy and Portugal) daily [58].

With reference to studies that have explored the status of adherence to dietary recommendations for specific countries, one study from Lebanon reported similar results, showing a higher adherence to the dietary guidelines for grains (about half children in the sample) and the lowest for fruit and vegetables (around 10–15%), while less than a quarter of the children met the recommendations on dairy, lean meat, and legumes [59]. Interestingly, differences by age groups were noted, with older participants being less adherent to the recommendations on vegetables, milk and dairy, lean meat, legumes, and grains than younger ones [59]. However, such trends have generally been reported quite consistently across various countries in the same Eastern Mediterranean region [60], concluding that a policy action is needed to improve the dietary habits of younger generations in this Mediterranean region.

Insufficient intake (up to 80%) of plant-food groups, including fruit, vegetables, and cereals, in Spanish children (6–7 years old) and adolescents (13–17 years old) was already reported in previous studies conducted over 20 years ago [61,62]. More recent available information confirmed a low percentage (less than 25%) of adolescents meeting recommendations for fruit and vegetables [63], while higher agreement was reported for dairy products, olive oil, and cereal products [64]. Data updated during the COVID-19 pandemic showed substantially unchanged trends, with the lowest adherence to dietary recommendations for vegetables, fruit, protein-rich foods and snacks, while higher agreement for olive oil and processed meat consumption [65]. Other recent reports, although not directly assessing adequacy in relation to dietary recommendations, confirmed that dairy products and cereals are the major contributors to daily energy intake, while fruit, vegetables, and also nuts and legumes are minor contributors [66]. These findings are consistent with those reported in the present study.

Concerning Italy, an early study conducted 25 years ago involving first to third year primary students showed that fruit (107 ± 100 g/day), vegetables (186 ± 74 g/day), and legumes (16 ± 29 g/day) were lower than recommended [67]. Similar findings from the same period were found in a sample of children of the same age, reporting average daily intakes of fruit (128 g), vegetables (161 g), and legumes (12 g) that were lower than recommended [68]. A subsequent study conducted on 8–9 year old Italian children confirmed that the average daily intakes of fruit (234 g/d), vegetables (134 g/d), and legumes (17 g/d) were lower than the nationally recommended ones [69]. Also, the consumption of whole grains in Italian children was shown to be significantly lower than the recommendations [70].

To our knowledge, no previous study has investigated adequacy in relation to dietary recommendations for children and adolescents in Portugal. However, a report from the National Food, Nutrition, and Physical Activity Survey showed an average low consumption of fruit and vegetables (only about one portion per day) in both Portuguese children and adolescents, and a more adequate consumption of dairy products, but only in children [71].

In the present study, some differences in diet quality by background characteristics have been found. Younger children reporting having 8–10 h adequate sleep duration, <2 h/day screen time, and medium/high physical activity displayed a better diet quality. Moreover, older respondents with a medium/high educational level, living with a partner, reported that their children had a better diet quality. There is consistent evidence in the scientific literature supporting the hypothesis that certain demographic, social, and cultural factors related to the parents affects the diet quality of their children in the countries involved in the present study [72,73,74,75,76]. Similarly, the overall lifestyle of children and adolescents is likely to include a cluster of healthy behaviors, including better dietary habits together with a more active lifestyle and better sleep hygiene [77,78,79]. Interestingly, the role of economic factors seems to be only secondary to children’s dietary choices. It may be possible that the relatively easy availability and affordability of plant-derived foods and other healthy food groups in the Mediterranean region could still be a strength to support healthy dietary habits [80]. Future studies are needed to provide more complete information on this matter to better identify the conceptualized framework.

The results presented in this study should be interpreted while taking into account certain limitations. The survey was conducted on a convenience sample of volunteers, which is not representative of the general national population. Moreover, although the participation rate was high, we have no information on non-responders or those who did not agree to join the study. Secondly, dietary habits are reported by the parents, which may lead to uncertainty concerning the validity of the answer, especially for adolescents, whose dietary habits may be less under the direct control of parents. Additionally, dietary habits are self-reported, leading to some potential additional biases, such as recall bias and social desirability. Finally, the adequacy in relation to dietary recommendations was based on national-specific dietary guidelines; hence, a direct comparison of percentage of adherence was not possible; moreover, differences in the types of food groups and frequencies considered make comparisons even more difficult, since the net consumption may have been similar but may not have resulted in the same level of adherence to the country-specific recommendation.

## 5. Conclusions

In conclusion, the results presented in this report confirm that plant-based food groups, including fruit, vegetables, legumes, and even (whole-grain) cereals, are underrepresented in the diets of Mediterranean children and adolescents. Moreover, the adequate consumption of other important dietary components, such as milk and dairy products, is rather disregarded, leading to substantially suboptimal diets and poor adequacy in relation to dietary guidelines. Additional studies could be useful to further investigate the reasons behind these dietary choices and the factors that could determine them. Various aspects concerning lifestyle behaviors, as well as psychosocial factors, could be potential targets to explore in future steps of the project. Additional and more complete information (such as food diaries) could also improve the quality of information concerning dietary exposure. Improving dietary adequacy among children and adolescents requires multifaceted strategies involving policy interventions, education, and community support. Governments and policy-makers can play a crucial role by implementing policies that promote the availability and affordability of healthy foods. Subsidies for fruits and vegetables, regulations on marketing unhealthy foods to children, and the promotion of school meal programs that follow the principles of the Mediterranean diet could be effective strategies.

## Figures and Tables

**Table 1:** Daily and weekly intake of food groups in children and adolescents from five Mediterranean countries.

	Spain (*n* = 411)	Egypt (*n* = 400)	Italy (*n* = 402)	Lebanon (*n* = 398)	Portugal (*n* = 400)	*p*-Value
Vegetables, *n* (%)						<0.001
Never	22 (5.4)	30 (7.5)	37 (9.2)	10 (2.5)	26 (6.5)	
1–2 portion/d	336 (81.8)	329 (82.3)	314 (78.1)	329 (82.7)	344 (86.0)	
≥3 portion/d	53 (12.9)	41 (10.3)	51 (12.7)	59 (14.8)	30 (7.5)	
Fruit, *n* (%)						<0.001
Never	34 (8.3)	19 (4.8)	26 (6.5)	3 (1.0)	11 (2.8)	
1–2 portion/d	319 (77.6)	316 (79.0)	296 (73.6)	319 (80.2)	298 (74.5)	
≥3 portion/d	58 (14.1)	65 (16.3)	80 (19.9)	76 (19.1)	91 (22.8)	
Cereals, *n* (%)						<0.001
Never	38 (9.2)	7 (1.8)	43 (10.7)	15 (3.8)	3 (1.0)	
1–2 portion/d	313 (76.2)	381 (95.3)	347 (86.3)	281 (70.6)	379 (94.8)	
≥3 portion/d	60 (14.6)	12 (3.0)	12 (3.0)	102 (25.6)	18 (4.5)	
Dairy, *n* (%)						<0.001
Never	112 (27.3)	94 (23.5)	62 (15.4)	56 (14.1)	80 (20.0)	
1–2 portion/d	210 (51.1)	272 (68.0)	261 (64.9)	181 (45.5)	237 (59.3)	
≥3 portion/d	89 (21.7)	34 (8.5)	79 (19.7)	161 (40.5)	83 (20.8)	
Meat, *n* (%)						<0.001
Never	88 (21.4)	16 (4.0)	17 (4.2)	7 (1.8)	15 (3.8)	
1–2 portion/w	230 (56)	259 (64.8)	197 (49.0)	246 (61.8)	109 (27.3)	
≥3 portion/w	93 (22.6)	125 (31.3)	188 (46.8)	145 (36.4)	276 (69.0)	
Legumes, *n* (%)						<0.001
Never	26 (6.3)	24 (6.0)	24 (6.0)	9 (2.3)	18 (4.5)	
1–2 portion/w	296 (72.0)	200 (50.0)	313 (77.9)	323 (81.2)	263 (65.8)	
≥3 portion/w	89 (21.7)	176 (44.0)	65 (16.2)	66 (16.6)	119 (29.8)	
Fish, *n* (%)						<0.001
Never	178 (43.3)	49 (12.3)	31 (7.7)	10 (2.5)	17 (4.3)	
1–2 portion/w	198 (48.2)	310 (77.5)	308 (76.6)	330 (82.9)	224 (56.0)	
≥3 portion/w	35 (8.5)	41 (10.3)	63 (15.7)	58 (14.6)	159 (39.8)	
Nuts, n (%)						<0.001
Never	167 (40.6)	217 (54.3)	151 (37.6)	45 (11.3)	173 (43.3)	
1–2 portion/w	205 (49.9)	155 (38.8)	215 (53.5)	294 (73.9)	183 (45.8)	
≥3 portion/w	39 (9.5)	28 (7.0)	36 (9.0)	59 (14.8)	44 (11.0)	
Whole grains, n (%)						<0.001
Never	181 (44.0)	111 (27.8)	104 (25.9)	117 (29.4)	54 (13.5)	
1–2 portion/w	167 (40.6)	162 (40.5)	149 (37.1)	183 (46.0)	148 (37.0)	
≥3 portion/w	63 (15.3)	127 (31.8)	149 (37.1)	98 (24.6)	198 (49.5)	
Sweets, *n* (%)						<0.001
Never	18 (4.4)	17 (4.3)	79 (19.7)	14 (3.5)	32 (8.0)	
1–2 portion/w	190 (46.2)	122 (30.5)	205 (51.0)	177 (44.5)	221 (55.3)	
≥3 portion/w	203 (49.4)	261 (65.3)	118 (29.4)	207 (52.0)	147 (36.8)	

**Table 2:** Mean YHEI scores across categories of children’s and adolescents’ background variables, total and by country.

	YHEI, Mean (SD)					
	Total	Spain	Egypt	Italy	Lebanon	Portugal
Age						
6–11 y	52.8 (11.5)	50.7 (10.4)	49.3 (10.6)	55.6 (11.3)	48.6 (9.6)	59.8 (11.7)
12–17 y	51.3 (11.7)	49.4 (10.6)	49.1 (12.4)	54.8 (11.8)	49.1 (11.6)	53.6 (11.4)
*p*-value	0.005	0.204	0.825	0.480	0.615	<0.001
Sex						
Male	51.6 (12.1)	49.9 (10.6)	48.4 (12.4)	54.5 (12.0)	48.9 (10.9)	56.3 (12.3)
Female	52.5 (11.7)	50.5 (10.4)	50.2 (10.6)	55.9 (11.1)	48.8 (10.3)	57.1 (11.6)
*p*-value	0.098	0.579	0.110	0.202	0.939	0.526
Sleep duration						
<8 h	49.7 (11.3)	49.8 (9.8)	47.7 (12.0)	54.6 (11.3)	48.4 (10.2)	50.7 (13.6)
8–10 h	52.8 (11.6)	50.6 (10.8)	49.9 (11.1)	55.3 (11.5)	49.5 (10.6)	57.6 (11.5)
>10 h	48.0 (11.7)	47.6 (10.9)	45.2 (14.7)	53.7 (13.9)	44.0 (10.9)	61.0 (9.8)
*p*-value	<0.001	0.366	0.081	0.809	0.025	<0.001
Screen time						
<2 h/day	53.3 (12.0)	50.3 (11.0)	50.2 (12.2)	56.8 (11.8)	49.6 (9.7)	57.4 (12.0)
2–4 h/day	50.6 (11.0)	50.6 (9.9)	48.5 (10.4)	53.3 (10.6)	48.1 (11.6)	54.7 (11.4)
>4 h/day	49.0 (11.7)	48.2 (10.6)	45.6 (10.8)	52.6 (12.1)	48.3 (9.8)	53.7 (14.6)
*p*-value	<0.001	0.405	0.080	0.007	0.391	0.118
Physical activity level						
Low	51.0 (11.22)	50.1 (10.0)	48.3 (11.3)	52.9 (11.0)	48.5 (10.6)	55.6 (11.2)
Medium	53.4 (12.1)	49.8 (11.2)	50.8 (11.6)	56.7 (11.3)	50.6 (11.1)	59.3 (12.6)
High	52.8 (12.0)	50.7 (11.0)	49.9 (11.7)	57.3 (11.9)	46.9 (9.4)	56.6 (12.4)
*p*-value	<0.001	0.807	0.188	0.001	0.041	0.050

**Table 3:** Mean YHEI scores across categories of parents’ background variables, total and by country.

	YHEI, Mean (SD)					
	Total	Spain	Egypt	Italy	Lebanon	Portugal
Age						
<44 y	49.8 (10.8)	51.8 (10.6)	47.5 (11.3)	50.8 (12.7)	48.5 (10.3)	53.4 (12.7)
≥45 y	52.6 (11.8)	49.5 (10.4)	49.4 (11.6)	55.4 (11.4)	49.3 (11.0)	56.9 (11.9)
*p*-value	<0.001	0.044	0.389	0.055	0.438	0.190
Sex						
Male	51.7 (11.2)	49.4 (10.7)	48.6 (10.8)	53.8 (10.7)	49.6 (10.2)	56.1 (11.6)
Female	52.3 (12.0)	50.9 (10.3)	49.5 (11.8)	56.2 (12.0)	48.3 (10.9)	57.1 (12.2)
*p*-value	0.246	0.142	0.489	0.042	0.216	0.394
Education level						
Low	47.6 (11.4)	49.8 (11.8)	44.1 (11.0)	50.63 (12.7)	45.4 (5.3)	47.6 (4.7)
Medium	52.0 (11.5)	50.3 (10.8)	48.7 (11.9)	53.7 (11.0)	50.0 (11.1)	54.5 (11.3)
High	52.5 (11.7)	50.4 (10.2)	51.2 (10.7)	57.8 (11.8)	48.9 (10.7)	58.8 (12.2)
*p*-value	0.001	0.961	0.004	0.001	0.685	<0.001
Family income						
<EUR 2000	51.6 (11.8)	49.4 (10.5)	49.9 (11.5)	53.3 (11.4)	50.9 (9.5)	55.0 (13.4)
EUR 2000–4000	52.6 (11.8)	51.3 (11.2)	49.6 (11.5)	54.8 (11.8)	47.6 (9.8)	57.5 (11.3)
>EUR 4000	51.1 (11.8)	50.2 (10.6)	50.0 (11.4)	58.0 (11.5)	49.1 (11.4)	56.6 (13.4)
*p*-value	0.100	0.455	0.819	0.061	0.149	0.190
Family status						
Live alone	50.8 (11.8)	50.8 (10.0)	48.6 (12.1)	52.3 (13.4)	48.1 (9.9)	55.5 (11.9)
Live with a partner	52.3 (11.7)	50.2 (10.5)	49.5 (11.5)	55.5 (11.3)	48.9 (10.6)	57.1 (11.9)
Live with others	47.2 (10.7)	46.7 (12.2)	43.4 (7.5)	54.6 (13.9)	47.5 (11.7)	47.8 (8.8)
*p*-value	0.003	0.502	0.258	0.203	0.797	0.055
Area of living						
Urban	51.9 (11.6)	50.3 (10.4)	49.4 (11.6)	55.1 (11.4)	48.8 (10.6)	56.6 (11.9)
Rural	52.3 (12.0)	49.8 (10.8)	48.8 (11.3)	55.6 (12.3)	49.0 (12.1)	57.0 (12.0)
*p*-value	0.653	0.692	0.642	0.723	0.950	0.745

## Data Availability

All data are available upon reasonable request due to privacy.
